# Distinct Genetic Structure Reflects Ploidy Level Differentiation in Newly Discovered, Extremely Small Populations of *Xanthocyparis vietnamensis* from Southwestern China

**DOI:** 10.3389/fgene.2021.733576

**Published:** 2021-11-01

**Authors:** Yuliang Jiang, Tsam Ju, Linda E. Neaves, Jialiang Li, Weining Tan, Yusong Huang, Yan Liu, Kangshan Mao

**Affiliations:** ^1^ Guangxi Key Laboratory of Plant Conservation and Restoration Ecology in Karst Terrain, Guangxi Zhuang Autonomous Region and Chinese Academy of Sciences, Guangxi Institute of Botany, Guilin, China; ^2^ Key Laboratory of Bio-Resource and Eco-Environment of Ministry of Education, College of Life Sciences, State Key Laboratory of Hydraulics and Mountain River Engineering, Sichuan University, Chengdu, China; ^3^ Fenner School of Environment and Society, Australian National University, Canberra, ACT, Australia; ^4^ Administration of Mulun National Nature Reserve of Guangxi, Huanjiang, China

**Keywords:** karst, Cupressaceae, microsatellites, flow cytometry, ploidy level, conservation genetics

## Abstract

Population genetic assessment is crucial for the conservation and management of threatened species. *Xanthocyparis vietnamensis* is an endangered species that is currently restricted to karst mountains in southwestern China and Vietnam. This rare conifer was first recorded in 2002 from northern Vietnam and then in 2013 from Guangxi, China, yet nothing is known about its genetic diversity nor ploidy level variation, although previous cytological study suggest that Vietnamese populations are tetraploids. There have been about 45 individuals found to date in Guangxi, China. Here, we genotyped 33 *X. vietnamensis* individuals using 20 newly developed, polymorphic microsatellite loci, to assess the genetic variability of its extremely small populations. The genetic diversity of *X. vietnamensis* (*H*_E_ = 0.511) was lower than that of two other heliophile species, *Calocedrus macrolepis* and *Fokienia hodginsii*, which have similar distribution ranges. This is consistent with the signature of a genetic bottleneck detected in *X. vietnamensis*. Although the population genetic differentiation coefficient across loci is moderate (*F*_ST_ = 0.125), STRUCTURE analysis revealed two distinct genetic clusters, namely the northern and southern population groups; DAPC analysis grouped the southern populations together in one cluster separate from the northern populations; AMOVA analysis detected a significant genetic differentiation between the two population groups (*F*_RT_ = 0.089, *p* < 0.05), and BARRIER analysis detected a genetic barrier between them. Moreover, we detected differentiation in ploidy level between northern and southern populations, sampled individuals from the former and the later are all diploid and tetraploid cytotypes with mean genome sizes of 26.08 and 48.02 pg/2C, respectively. We deduced that heterogeneous geomorphology and historical events (e.g., human deforestation, Quaternary climate oscillations) may have contributed to population fragmentation and small population size in *X. vietnamensis*. Considering both genetic and ploidy level differentiation, we propose that two different management units (northern and southern) should be considered and a combination of *in situ* and *ex situ* conservation measures should be employed to preserve populations of this endangered species in southwestern China in the light of our findings.

## Introduction

Understanding the level and distribution of genetic variation has implications for the conservation of species and ecosystems (Bruford et al., 2017). Measures of genetic diversity are important elements for estimating species fitness and the potential for population persistence (Reed and Frankham, 2003). Generally, small population size and low gene flow among populations are associated with low genetic diversity of a species (Hamilton, 2009). Populations with low genetic diversity have a limited ability to adapt to, and survive environmental changes (Jump et al., 2009; Weeks et al., 2011), and active conservation is often needed for such populations. Thus, assessment of genetic variability is necessary for designing conservation programmes for endangered species (Yamamoto et al., 2017; Chung et al., 2020).

Many species of conservation concern are endemic, fragmented, and have limited gene flow and high level genetic differentiation among populations, which are generally attributed to cyclical climatic changes, complex geological history, and longstanding influence of human activities (González-Martínez et al., 2010; Liao et al., 2015; Li et al., 2020). Usually, high levels of endemism suggests *in situ* long-term survival, differentiation, and speciation (Tang 2015). For example, endangered relict plants are mostly paleoendemic, confined to a particular geographic area and, have typically been isolated for quite a long time (Hobohm, 2014). Their current distribution represents the remnants of a much larger paleo-distribution. It is a general inclination to treat endemic species as high-priority conservation targets, but conservation efforts are often faced with a dilemma (Groves, 2003; Edmands, 2007). Fragmentation of habitats is usually accompanied by reductions in population size, genetic isolation, increased inbreeding within populations, and decreases in genetic diversity (Aguilar et al., 2008). Hence, habitat fragmentation is considered one of the biggest threats to the survival of species with small and isolated populations, especially in the context of increased intensity of human activities all over the world (Hamrick, 2004; Liao et al., 2015). It is imperative to investigate the effect of habitat fragmentation on endangered relict plants.

*Xanthocyparis vietnamensis* Farjon & Hiep of the Cupressaceae family, was first described from the remnants of karst forests in the northern Vietnamese border in 2002, where mountainous regions were once inaccessible (Averyanov et al., 2002; Farjon et al., 2002). In recent decades, scattered individual trees of this species have been found in karst landscape in the northern and southern border counties of the Guangxi Zhuang Autonomous Region in southwestern China (Meng et al., 2013; Jiang et al., 2021). Populations in China and Vietnam are small in size (each containing 1–50 individuals) and inhabit isolated tops of steep and narrow mountain ridges, with low levels of reproduction under natural conditions (Averyanov et al., 2002; Averyanov et al., 2013; Jiang et al., 2021). According to the evaluation criteria from the 2013 International Union for Conservation of Nature and Natural Resource Red list, *X. vietnamensis* is classified as an endangered species (Thomas, 2013).

Although previous studies suggested that *X. vietnamensis* was sister to the Alaska Cedar (*Callitropsis nootkatensis*) and hence were placed into a genus that comprised of only these two species (Little et al., 2004; Little, 2006), another study placed *X. vietnamensis*, the Alaska Cedar, the New World Cypresses (*Hesperocyparis*) and the Old World Cypresses (*Cupressus sensu strico*) into a single genus (*Cupressus sensu lato*) (Christenhusz et al., 2011). However, recent phylogenetic and phylogenomic studies suggest that *X. vietnamensis* is basal, in a clade also containing the Alaska Cedar as sister to a monophyletic clade comprising all of the New World Cypresses (*Hesperocyparis*) (the HCX clade) (Mao et al., 2010; Mao et al., 2019); the HCX clade is then basal to a clade where the Old World Cypresses (*Cupressus sensu strico*) is sister to junipers (*Juniperus*) (Mao et al., 2019). Within the HCX clade, *X. vietnamensis* diverged from the other species around late Eocene (Mao et al., 2019). Although no fossil evidence exists for *Xanthocyparis* (*sensu stricto*), it is high likely that it has survived a long history of environmental changes since late Eocene, and high likely that the species had a considerably wider distribution in warmer geological periods. Hence, this recently discovered, endangered conifer merits further investigations to aid its conservation.

Recently, a ploidy screening analyses of Cupressaceae plants suggested that *X. vietnamensis* is tetraploid with a genome size of 44.60 pg/2C, which is unexpected considering the generally low frequency of polyploidy in extant gymnosperms (Farhat et al., 2021). However, this is based on an analysis of eight individuals collected from Vietnam. Previous studies had reported the natural intraspecific variation of ploidy level in a few gymnosperm species, such as *Juniperus chinensis* and *J*. *sabina* (Farhat et al., 2019). Based on our morphological observation, the scale-like leaves in *X. vietnamensis* individuals from northern Guangxi are always much smaller than those from southern Guangxi (Supplementary Figure S1). Since tetraploids usually possess bigger leaf size than diploids (Zhang et al., 2019), here we propose a novel hypothesis: *X. vietnamensis* populations in Guangxi have two cytotypes, namely diploids and tetraploids. Extensive studies are now needed to reveal the ploidy level variation within and between populations of *X. vietnamensis*.

Southwestern China and northern Vietnam, with their warm, humid climates and diverse topographies, have been identified as long-term climatically stable refugia likely to preserve ancient lineages, meaning these refugia may be prioritized for the conservation of relict plants (Tang et al., 2018). These regions have drawn significant attention from researchers. Conservation genetic studies have been conducted for many threatened plant species, such as *Calocedrus macrolepis* and *Fokienia hodginsii* (Liao et al., 2015; Yin et al., 2021), yet no population genetic survey has been done for *X. vietnamensis*. Characterizing the genetic variation in populations of *X. vietnamensis* is essential for effective conservation. Therefore, we used microsatellite markers to assess the levels of genetic diversity and genetic bottleneck of recently discovered populations of *X. vietnamensis* in southwestern China, as well as genetic differentiation and gene flow among isolated populations. We also conduct an assessment of genome size and ploidy level to understand their ploidy level variation. Our aims were to: 1) reveal the current level and distribution of genetic variation across sampled sites (individuals) in Guangxi, China, 2) identify ploidy level variation of these population and test the connection between patterns of genetic and ploidy level variations, 3) detect the effects of habitat fragmentation and isolation on genetic characteristics, and 4) provide useful insights for optimal conservation strategies.

## Materials and Methods

### Sample Collection and DNA Extraction

**FIGURE 1:**
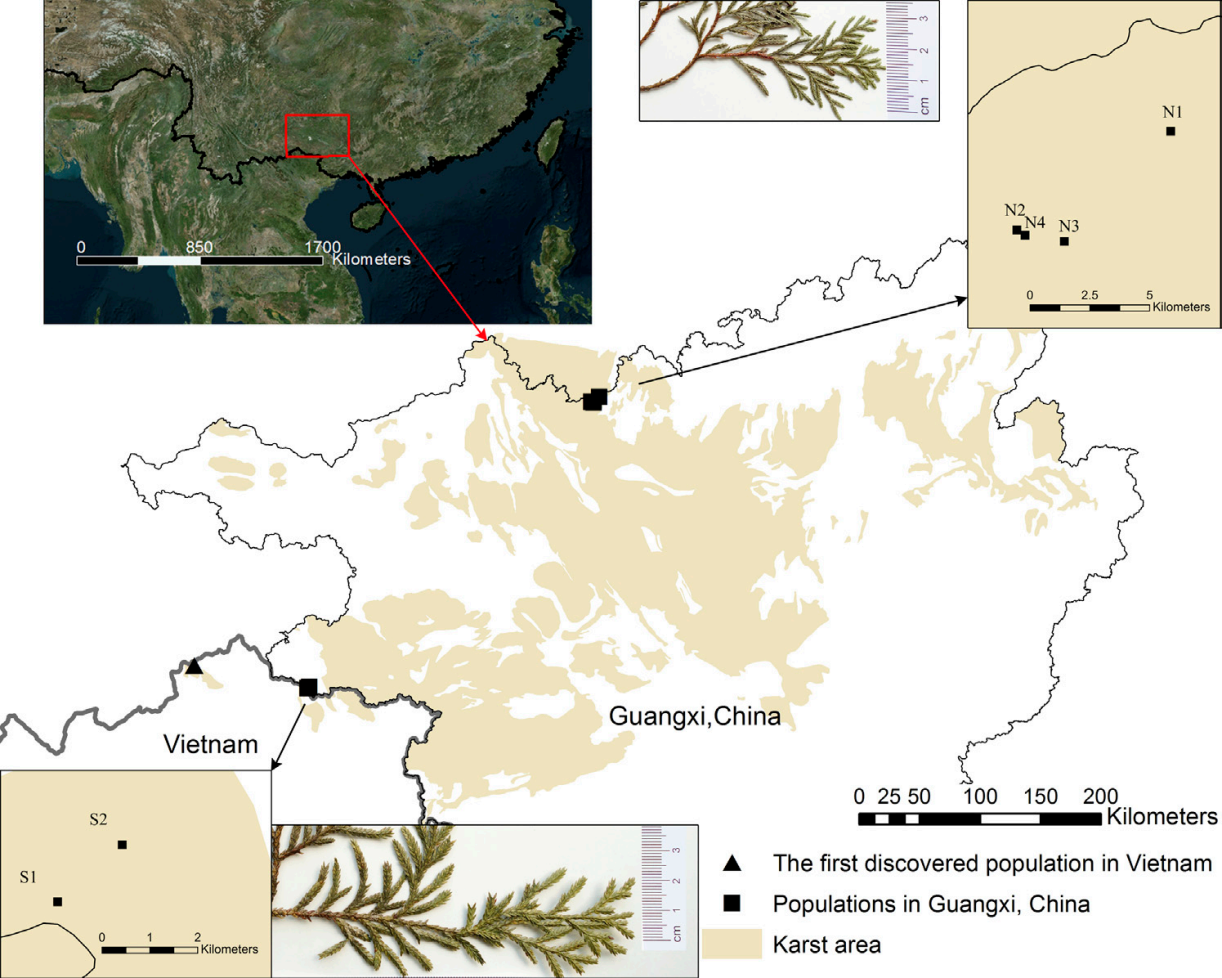
Current distribution and leaf appearance of *Xanthocyparis vietnamensis*in Guangxi, China and the first discovery site in Vietnam. Population site codes: N1, N2, N3, N4, S1, S2. Insets in top middle and bottom middle show the sizes of the scale-like leaves of individuals from northern and southern Guangxi, respectively. All the karst landforms in Guangxi and partial karst landforms in the other regions are showed by the yellow marker. See a direct comparison of scale-like leaf size between individuals from northern and southern Guangxi in Supplementary Figure S1.

During our field survey of *X. vietnamensis* in Guangxi, China from 2013 to 2020, there were eight karst mountains in Guangxi where this species was found. Five and three mountains were located in the northern and southern border of Guangxi respectively (Figure 1; Table 1). Each mountain had a small number (1–20) of wild individuals growing on or near steep slopes, and the total number of individuals was approximately 45. Fresh leaves of 33 individuals were collected from six sites (mountains), immediately dried in silica gel and stored at room temperature prior to DNA extraction. Additionally, fresh and young leaves off our mature individuals were collected from each of the four sites with more than four individuals (Table 2) and kept frozen at −80°C until analyzed for ploidy level determination.

**TABLE 1:** Distribution sites of *Xanthocyparis vietnamensis* in Guangxi, southwestern China.

Locality	Code	Latitude	Longitude	Altitude (m)	Sample size
North					
Tianwanshan mountain, Mulun National Nature Reserve	N1	25°09′25″	108°00′25″	724	1
Baxia mountain, Mulun National Nature Reserve	N2	25°07′09″	107°56′57″	814	1
Mingli mountain, Mulun National Nature Reserve	N3	25°06′54″	107°58′03″	820	14
Mingwei mountain, Mulun National Nature Reserve	N4	25°07′01″	107°57′09″	850	7
Shisantang, Nandan county	—	25°01′43″	107°47′17″	814	0
South					
Guanhua mountain, Laohutiao Provincial Nature Reserve	S1	22°59′56″	105°50′59″	1,240	6
Nongwai mountain, Laohutiao Provincial Nature Reserve	S2	22°59′15″	105°50′15″	1,264	4
Meilin mountain, Laohutiao Provincial Nature Reserve	—	22°59′12″	105°50′29″	1,244	0
				Total	33

**TABLE 2:** Estimation of genome size and ploidy level in individuals of *Xanthocyparis vietnamensis*.

Site	Individual	2C DNA (pg ± SD)	Ratio (±SD)	Ploidy level	Calibration standard
N3	N3-5	26.62 ± 0.14	0.51 ± 0.003	2*x*	I
	N3-8	26.39 ± 0.49	0.51 ± 0.009	2*x*	I
	N 3–11	25.42 ± 0.31	0.49 ± 0.006	2*x*	I
	N 3–14	25.63 ± 0.40	0.49 ± 0.008	2*x*	I
N4	N 4–2	25.95 ± 0.22	0.50 ± 0.004	2*x*	I
	N 4–4	26.50 ± 0.18	0.51 ± 0.003	2*x*	I
	N 4–5	25.47 ± 0.26	0.49 ± 0.005	2*x*	I
	N 4–7	26.63 ± 0.21	0.51 ± 0.004	2*x*	I
S1	S 1–1	50.48 ± 0.67	2.35 ± 0.031	4*x*	II
	S 1–2	49.05 ± 0.32	2.28 ± 0.015	4*x*	II
	S 1–3	48.59 ± 0.40	2.26 ± 0.018	4*x*	II
	S 1–4	47.40 ± 1.25	2.20 ± 0.058	4*x*	II
S2	S 2–3	46.59 ± 0.27	2.17 ± 0.013	4*x*	II
	S 2–4	47.08 ± 1.05	2.19 ± 0.049	4*x*	II
	S 2–5	47.90 ± 0.36	2.23 ± 0.017	4*x*	II
	S 2–6	47.04 ± 0.37	2.19 ± 0.017	4*x*	II

Ratio: Ratio of 2°C peak positions between the sampled individual and calibration standard. Calibration standard (I) *Pseudolarix amabilis* (II) *Cupressus funebris.*

Sampling was limited because *X. vietnamensis* individuals were rare, and some individuals or sites were inaccessible (Table 1). Genomic DNA was extracted from the leaf tissue of all sampled individuals following the cetyltrimethylammonium bromide (CTAB) method (Doyle and Doyle, 1990).

### Short Sequence Repeat Marker Discovery, Screening, and Data Production

Total RNA was extracted from fresh leaves of one *X. vietnamensis* individual for Illumina sequencing (Mao et al., 2019). The cDNA library was sequenced using the Illumina HiSeq 2000 system at Novogene (Beijing, China). Sequences were filtered, and clean reads were assembled *de novo* using Trinity v.2.8.5 (Grabherr et al., 2011). Short sequence repeat (SSR) of motifs were identified using the Perl script MISA (Thiel et al., 2003). Primer3 v.2.3.6 was used to design primer pairs for the detected markers (Rozen and Skaletsky, 2000; Untergasser et al., 2012). A total of 300 SSR loci were applied for pilot screening of polymorphic makers based on 16 individuals, and these polymorphic loci were then employed to detect genetic variation for all sampled individuals.

PCR amplification was performed in 25 μl reactions containing 50 ng of genomic DNA, 12.50°μl of 2× PCR buffer, 300 μM of each dNTP, 0.3 μM of primer pairs, and 1.25 U of Taq DNA polymerase (all from Vazyme Biotech, Nanjing, China). The amplification program was performed as follows: initial denaturation at 98°C for 3°min; followed by 40 cycles at 98°C for 10 s, annealing temperatures (see Supplementary Table S1) of the primer pair for 30°s, 72°C for 45°s; and a final extension step at 72°C for 10 min. Each forward primer was labeled with either FAM, HEX, and TAMRA at the 5′ extremity to allow fragment detection (Supplementary Table S1). Amplified products were inspected in a 1% agarose gel and electrophoresed on the ABI PRISM 3100 genetic analyzer (Applied Biosystems, Foster City, CA, United States). The microsatellite genotype at each locus, for each individual was determined using GeneMapper v.4.1 (Soft Genetics, State College, PA, United States). The codominant SSR data were analyzed using Cervus3.0 software (Kalinowski et al., 2007) to detect genotyping errors at each locus, such as stuttering, large allele dropouts and null alleles. When the proportion of null alleles at a locus is greater than 40% (*F*_(Null)_ > 0.4), it was deemed to contain excessive null alleles. Loci were tested for natural selection using BayeScan (Foll and Gaggiotti, 2008), and the loci under selection were excluded for further population genetic analyses.

### Genetic Diversity and Differentiation

The following measures of genetic diversity were calculated for each locus, population, and geographic group (south and north) using GenAlEx v.6.5 (Peakall and Smouse, 2012): number of different alleles (*A*), number of effective alleles (*A*_E_), Shannon’s diversity index (*I*), observed heterozygosity (*H*_O_), expected heterozygosity (*H*_E_), inbreeding coefficient (*F*_IS_), and genetic differentiation coefficients between groups (*F*_ST_) per locus. The statistical significance of the deviation of loci from Hardy-Weinberg equilibrium (*HWE*) and the linkage disequilibrium test between two loci were assessed by the Markov chain method in Genepop v.4.7 (Rousset, 2008). Sequential Bonferroni correction was used to determine significance level at *p* < 0.05 (Rice, 1989). We also implemented hierarchical analysis of molecular variance (AMOVA) (Excoffier et al., 1992) in GenAlEx v.6.5 to calculate the level of genetic variation and differentiation among geographic groups, among populations within groups, and among individuals within populations based on *F*-statistics (Wright, 1978), using data of the four populations with more than one sample. The significance of the differences was tested using permutation procedures (999 replicates).

The bottleneck test for population was estimated using the M-ratio method (Garza and Williamsion, 2001), which is the ratio of the total number of alleles to the overall range of allele size. We assumed a microsatellite mutation rate per locus per generation of 10^−4^ and pre-bottleneck effective population size of 400 (*θ* = 4*N*_e_ *μ* = 0.16) and 4,000 (*θ* = 1.6) to estimate the M-ratio. The mean size of non-single-step mutations and the percentage of mutations larger than a single step were set to 3.5 and 0.1, respectively (Garza and Williamsion, 2001). The M-ratio (*M*_0_) estimated using the program M_P_Val was compared to the 95% critical M-ratio (*M*_c_) estimated using the Critical_M program. We assumed that the population experienced a significant bottleneck if *M*_0_ < *M*_c_ when *N*_e_ = 4,000, and a moderate bottleneck if *M*_0_ < *M*_c_ when *N*_e_ = 400.

### Population Structure and Gene Flow

The Bayesian clustering method in STRUCTURE v.2.3.4 (Pritchard et al., 2000) was used to assess the genetic clustering of sampled *X. vietnamensis* populations. This analysis was run for 10 independent runs per *K* value (1–10) with a burn-in period of 50,000 iterations and 100,000 Markov chain Monte Carlo (MCMC) iterations, using the admixture (allele frequencies correlated) model. Structure Harvester (Earl, 2012) was used to visualize the best *K* value based on delta *K* and maximum log likelihood L(*K*) (Evanno et al., 2005). Discriminant analysis of principal components (DAPC) was performed to identify the differentiated subpopulations using the R package Adegenet (Jombart, 2008; Jombart et al., 2010). The number of clusters was set at 10. The microsatellite data was transform into principal components (PCs) using the function *find.clusters*, retaining all the PCs in the analysis. The Bayesian information criterion (BIC) was used to infer the optimal number of clusters (Jombart et al., 2010). The functions *a.score* and *optim.a.score* were applied to identify the optimal number of PCs to be retained. The function *dapc* performed discriminant analysis using the optimal number of PCs and four eigenvalues were retained.

MIGRATE-n (version 3.3.1) was used to estimate the mutation-scaled effective population size *Θ* (4*N*_e_ *μ*; *N*_e_, effective population size; *μ*, mutation rate per locus per generation), mutation-scaled migration rate *M* (*m*/*μ*; *m*, historical migration rate per generation), and the number of migrants *N*_m_ (*N*_e_ *m*) between the two geographic groups (Beerli, 2006). We used a uniform prior distribution to estimate *Θ* (rang 0–80), *M* (range 0–300), and reported the mean and 97.5% confidence intervals for *Θ* and *M*. The starting values for *Θ* and *M* were estimated using the *F*_ST_. Monmonier’s maximum difference algorithm implemented in BARRIER v.2.2 (Manni et al., 2004) was used to identify potential barriers to gene flow among populations. Nei’s distance matrices, used in determining the barriers, were produced by bootstrapping over loci with Microsatellite Analyser v.4.05 (Dieringer and Schlötterer, 2003).

### Genome Size Assessment and Ploidy Level Determination

Genome size was assessed for 16 individuals of *X.vietnamensis* (Table 2). Relative nuclear DNA content of the individuals was estimated by propidium iodide (PI) flow cytometry using fresh leaves (Bourge et al., 2018; Farhat et al., 2021). We adopted the nuclei isolation buffer “Galbraith’s” (Galbraith et al., 1983), and samples of *Cupressus funebris* (2*n* = 22 and 2C = 21.52 pg DNA) (Hizume et al., 2001) and *Pseudolarix amabilis* (2*n* = 44 and 2C = 52.2 pg DNA) (Zonneveld, 2012) were selected as the internal calibration standards. For each sample, about 1 g of fresh leaves were placed into a glass Petri dish. Then leaves were chopped using a razor blade in 4 ml of 4°C cold buffer. The nuclear suspension was filtered through 37 or 48 μm nylon mesh. RNase A was added to the suspension to prevent staining of double-stranded RNA. Cell nuclei were stained with 100 μg/ml PI, and incubated in the dark for 30 min at 4°C before analysis by flow cytometry.

BD FACSCalibur™ Flow Cytometer (BD Biosciences, United States) equipped with a solid-state laser for PI excitation was used to detect DNA content (2C value). Three replicates were run per individual, recording at least 10,000 particles (including nuclei and fragments) per replicate. The data analysis was performed in BD CellQuest™ Pro (version 6.1). The resulting histograms were analyzed with ModFit LT 4.1.7 software. When CV values of G0/G1 peaks were below 5%, the analyses saved. The determination of ploidy level was based on the relative nuclear DNA contents and published chromosome counts (Farhat et al., 2021). The databases were accessed on August 11, 2021: Kew Plant DNA C-values database (http://data.kew.org/cvalues) and Index to Plant Chromosome Number (IPCN) (http://legacy.tropicos.org/Project/IPCN).

## Results

### Genetic Diversity and Genetic Structure

Twenty microsatellite markers were polymorphic in the *X. vietnamensis* populations analyzed in this study (Supplementary Table S1). No loci with excessive null allele and no loci under natural selection were detected. A total of 96 alleles were detected, ranging from two to ten alleles per locus, with an average of 4.8 (Table 3). The observed heterozygosity (*H*_O_) and expected heterozygosity (*H*_E_) per locus ranged from 0.156 to 0.940 and from 0.219 to 0.839, respectively. The inbreeding coefficient (*F*_IS_) per locus ranged from −0.379 to 0.575. Five loci displayed significant deviations from Hardy-Weinberg equilibrium and 10% locus pairs showed evidence of linkage disequilibrium after sequential Bonferroni correction. All loci were retained for further analysis. The genetic differentiation between the geographic groups (*F*_ST_) varied among loci, ranging from 0.008 to 0.450 (Table 3).

**TABLE 3:** Genetic variation of 20 microsatellite markers used in this study in the population of *Xanthocyparis vietnamensis* in Guangxi, China.

Locus	*A*	*H*_O_	*H*_E_	*F*_IS_	*F*_ST_
seq89	4	0.364	0.307	−0.184	0.149
seq2225	3	0.394	0.577	0.317	0.356
seq24651	10	0.818	0.839	0.025	0.059
seq5172	6	0.606	0.774	0.217	0.047
seq11372	4	0.727	0.528	−0.379	0.024
seq12084	7	0.419	0.494	0.152^a^	0.049
seq13330	7	0.600	0.760	0.211^a^	0.099
seq17433	6	0.500	0.476	−0.051	0.085
seq19571	5	0.303	0.387	0.216	0.170
seq20427	5	0.273	0.527	0.483^a^	0.181
seq22472	3	0.182	0.219	0.168	0.194
seq22970	3	0.281	0.512	0.451	0.450
seq24226	4	0.455	0.680	0.332^a^	0.138
seq25166	9	0.677	0.767	0.117	0.101
seq25799	4	0.156	0.368	0.575^a^	0.061
seq27386	4	0.281	0.294	0.043	0.008
seq30772	4	0.531	0.448	−0.186	0.051
seq31782	2	0.364	0.298	−0.222	0.133
seq33197	2	0.212	0.236	0.099	0.108
seq34736	4	0.939	0.740	−0.270	0.030
Mean ± SE	4.8 ± 0.5	0.454 ± 0.049	0.511 ± 0.044	0.106 ± 0.059	0.125 ± 0.025

A, Number of alleles; *H*_O_, observed heterozygosity; *H*_E_, expected heterozygosity; *F*_IS_, inbreeding coefficient; *F*_ST_, genetic differentiation between southern and northern groups.

a Significant deviation from *HWE* after sequential Bonferroni correction.

The genetic diversity estimates at the population and group levels are summarized in Table 4. N3 had the highest *H*_E_ (0.439), and N4 had the lowest *H*_E_ (0.373) at the population level. At the group level, the north had a slightly higher *H*_E_ (0.469) than the south (0.438). The M-ratio test for the *X. vietnamensis* populations detected a significant bottleneck (*M*_0_ = 0.7737; *M*_c_ = 0.7883 when the pre-bottleneck *N*_e_ = 4,000, *p* = 0.0326; *M*_c_ = 0.8693 when the pre-bottleneck *N*_e_ = 400, *p* = 0.0002).

**TABLE 4:** Comparison of genetic diversity of *Xanthocyparis vietnamensis* populations of different levels in Guangxi, China.

		*n*	*A*	*A*_E_	*I*	*H*_O_	*H*_E_	*F*_IS_
Population level	N3	14	3.9	2.2	0.841	0.368	0.439	0.136
	N4	7	2.7	1.9	0.646	0.388	0.373	−0.052
	S1	4	2.7	2.1	0.732	0.563	0.427	−0.324
	S2	6	2.5	2.0	0.680	0.617	0.411	−0.468
	Mean	7.8	3.0	2.1	0.725	0.484	0.413	−0.177
Region level	North	23	4.3	2.4	0.898	0.392	0.469	0.150
	South	10	3.0	2.2	0.767	0.594	0.438	−0.346
	Mean	16.5	3.7	2.3	0.833	0.493	0.454	−0.098
Species level		33	4.8	2.5	0.992	0.454	0.511	0.106

*n*, Number of samples; *A*, number of different alleles; *A*_E_, number of effective alleles; *I*, Shannon’s information index; *H*_O_, observed heterozygosity; *H*_E_, expected heterozygosity; *F*_IS_, inbreeding coefficient.

**FIGURE 2:**
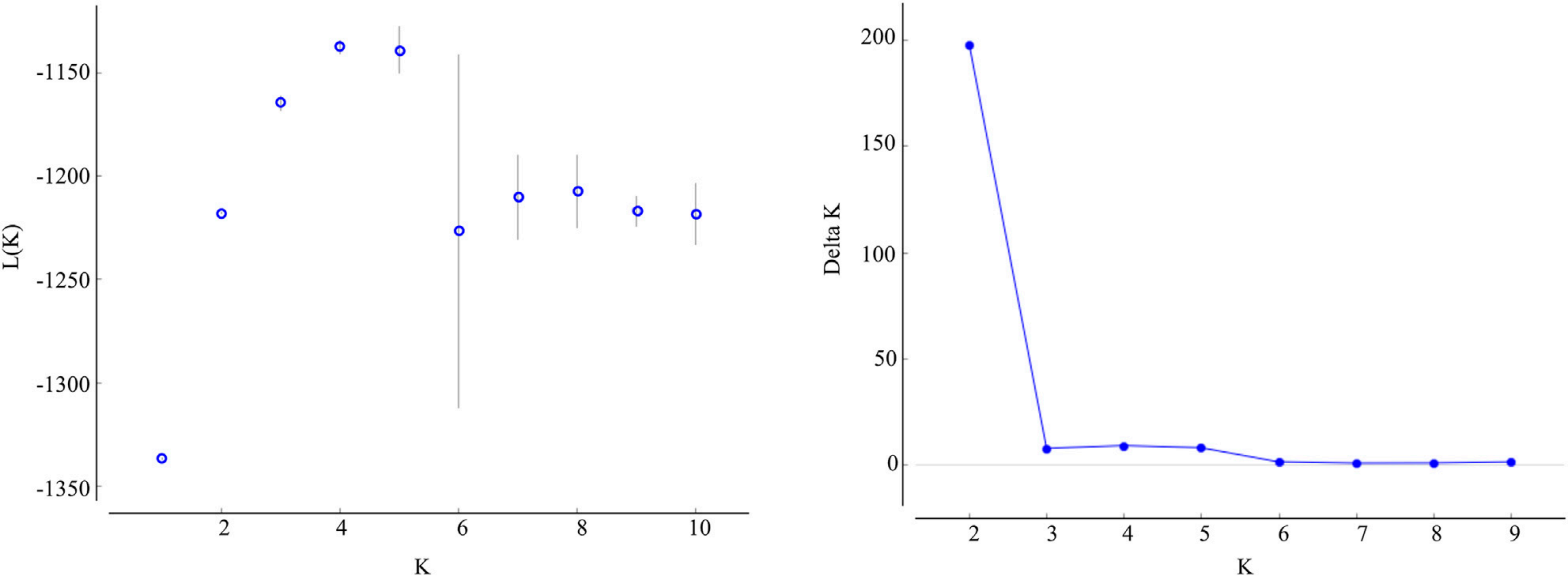
Values of the log likelihood of the data(L(K)) (mean ± SD), as a function of the number of clusters (K) resulting from the simulation in the STRUCTURE method, and delta K based on the rate of change of L(K) between successive K values.

**FIGURE 3:**
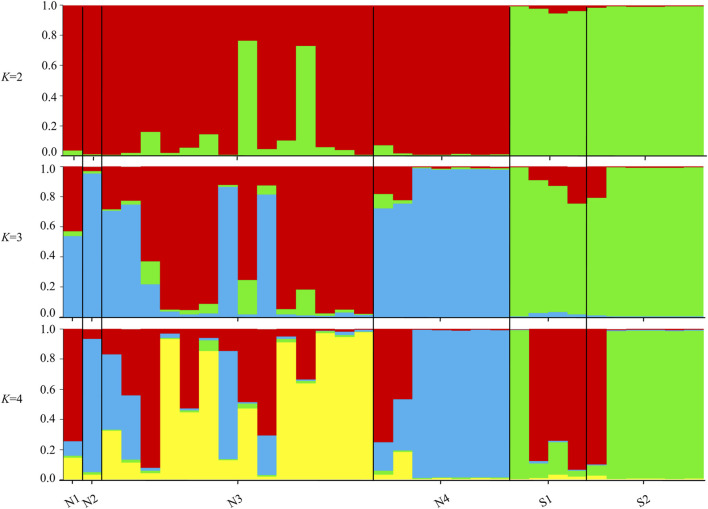
Histograms of the STRUCTURE assignment test for all individuals at *K* = 2, 3, 4. Labels on the x-axis reference to sampling site and individual (Northern sites = N1, N2, N3, N4 and Southern sites = S1, S2).

**FIGURE 4:**
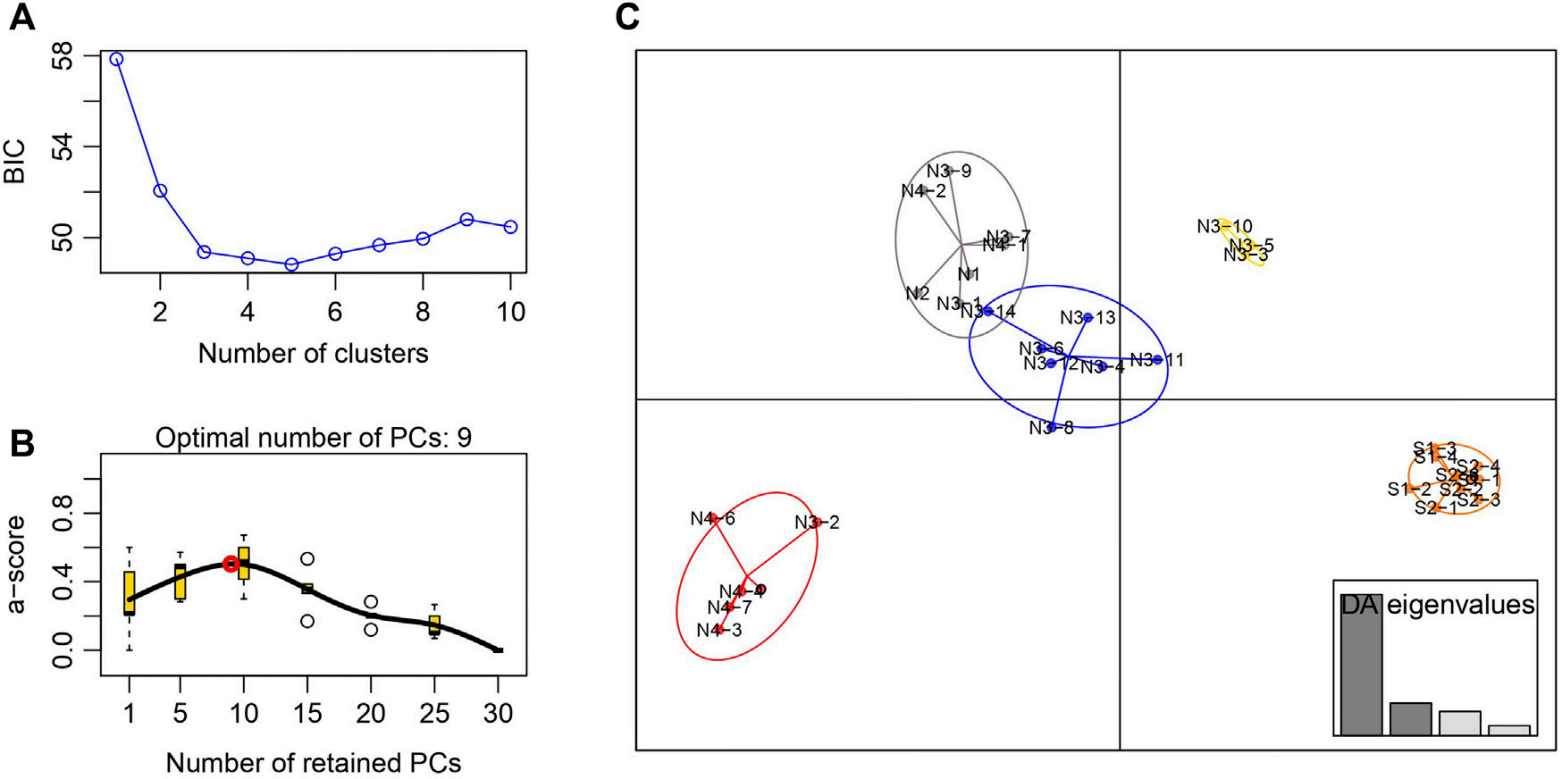
Discriminant analysis of principal components of the microsatellite data for *Xanthocyparis vietnamensis* populations from Guangxi, China **(A)** Value of BIC versus number of clusters **(B)** A-score optimisation—spline interpolation **(C)** Scatter plots of the discriminant analysis of principal components. Each circle represents a cluster and each dot represents an individuals. DAPC assignment plot based on four discriminant functions. Eigenvalues are displayed in inset.

The STRUCTURE results showed that the L(*K*) continued to increase up to *K* = 4, and the *K* = 2 model received the highest delta *K* value (Figure 2). Histogram showing the results of *K* = 2 model revealed clear differences between the northern and southern populations (Figure 3). When *K* = 3 and 4, the N4 population separated from other northern populations, resulting in a slight signature of separation within the northern populations (Figure 3). The BIC curve in the DAPC analysis suggested that the optimal number of clusters was five (Figure 4A). The southern populations (S1 and S2) were assigned to a single cluster while the northern populations were assigned to the other four (Figure 4C). Specifically, individuals of N3 were assigned to four different clusters, and N4 were assigned to two separate clusters (Figure 4C).

### Population Genetic Differentiation and Historical Gene Flow

The genetic differentiation coefficient average across loci (*F*_ST_ = 0.125, SE = 0.025) suggested a moderate level of differentiation between the northern and southern population groups (Table 3). AMOVA showed more genetic variation was partitioned between the northern and southern population groups (6%) than among populations within groups (3%); most genetic variation was found within individuals (91%). Genetic differentiation between the northern and southern population groups (*F*_RT_ = 0.089) was statistically significant (*p* < 0.05), while the genetic differentiation among populations and among individuals were not statistically significant (Table 5).

**TABLE 5:** Analysis of molecular variance (AMOVA) results of the four sub-populations (N3, N4, S1, S2).

Source of variation	*df*	*SS*	*MS*	*Est. Var*	*V*%	*F*-statistics
Among groups	1	27.481	27.481	0.706	6	*F*_RT_ = 0.089^a^
Among populations	2	16.437	8.218	0.310	3	*F*_SR_ = 0.043
Among individuals	27	103.565	3.836	0.000	0	*F*_TS_ = −0.444
Within individuals	31	309.000	9.968	9.968	91	

*df*, degrees of freedom; *SS*, sum of squares; *MS*, mean square; *Est. Var*., estimated variation; *V*%, percent variation; *F*_RT_, differentiation among groups; *F*_SR_, differentiation among populations within groups; *F*_TS_, differentiation among individuals within populations.

a *p* < 0.05, 999 permutations.

**FIGURE 5:**
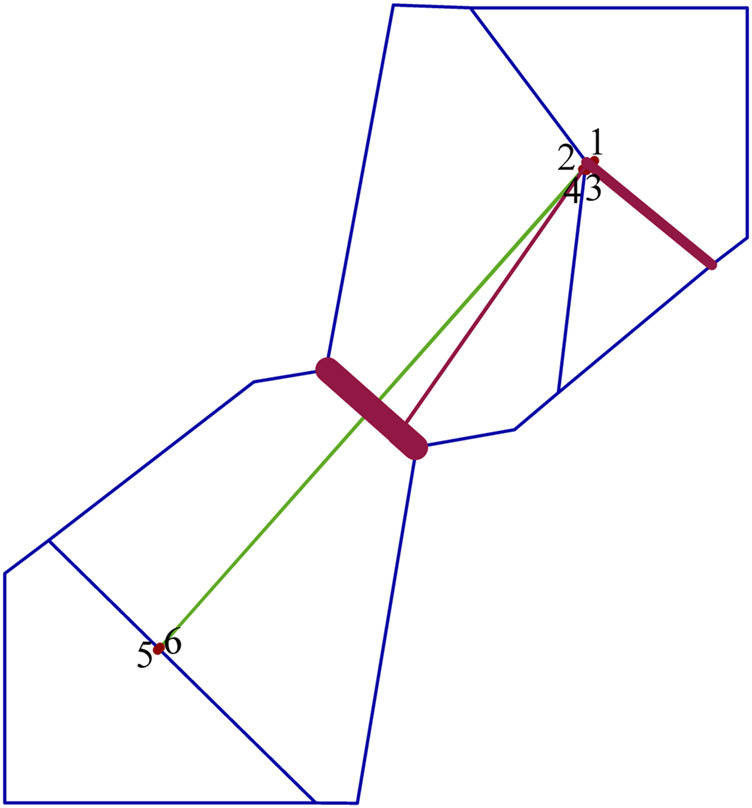
Delaunay triangulation (in green) and Voronoï tessellation (in blue) of the barrier analyses for *Xanthocyparis vietnamensis*. Barriers (in dark red) are detected with bootstrap values of 100 replicates using Nei’s standard genetic distance. Red points correspond to the six sampling sites (1, N1; 2, N2; 3, N3; 4, N4; 5, S1; 6, S2).

The mean *Θ* values for northern and southern populations were 0.896 and 0.644, respectively. Asymmetric gene flow between north and south populations was detected, with the number of migrant individuals per generation (*N*_m_) from south to north (0.770) being less than from north to south (2.008) (Table 6). The BARRIER analysis revealed a well-supported genetic and/or biogeographic barrier (with bootstrap support value of 75%) that separating the northern and southern populations (Figure 5).

**TABLE 6:** Summarized results of the MIGRATE-n analyses.

Parameter	Mean	97.5% confidence interval	*N*_m_	Autocorrelation	ESS
*Θ*_1_	0.89641	(0, 2.13)	—	0.92984	3053.87
*Θ*_2_	0.64430	(0, 1.92)	—	0.96254	2447.63
*M*_2->1_	3.434	(0, 8.20)	0.770	0.97101	2581.07
*M*_1->2_	12.467	(6.00, 18.60)	2.008	0.98328	2945.87
Ln[Prob(D|G)]	—	—	—	0.94404	4810.37

*Θ*, mutation-scaled effective population size; *M*, mutation-scaled migration rate; *N*_m_, the number of migrants estimated for the north (1) and south (2) population clusters. The included autocorrelation and the estimated sample sizes (ESS) values were used to calculate convergence during the runs.

### Genome Size and Ploidy Level

**FIGURE 6:**
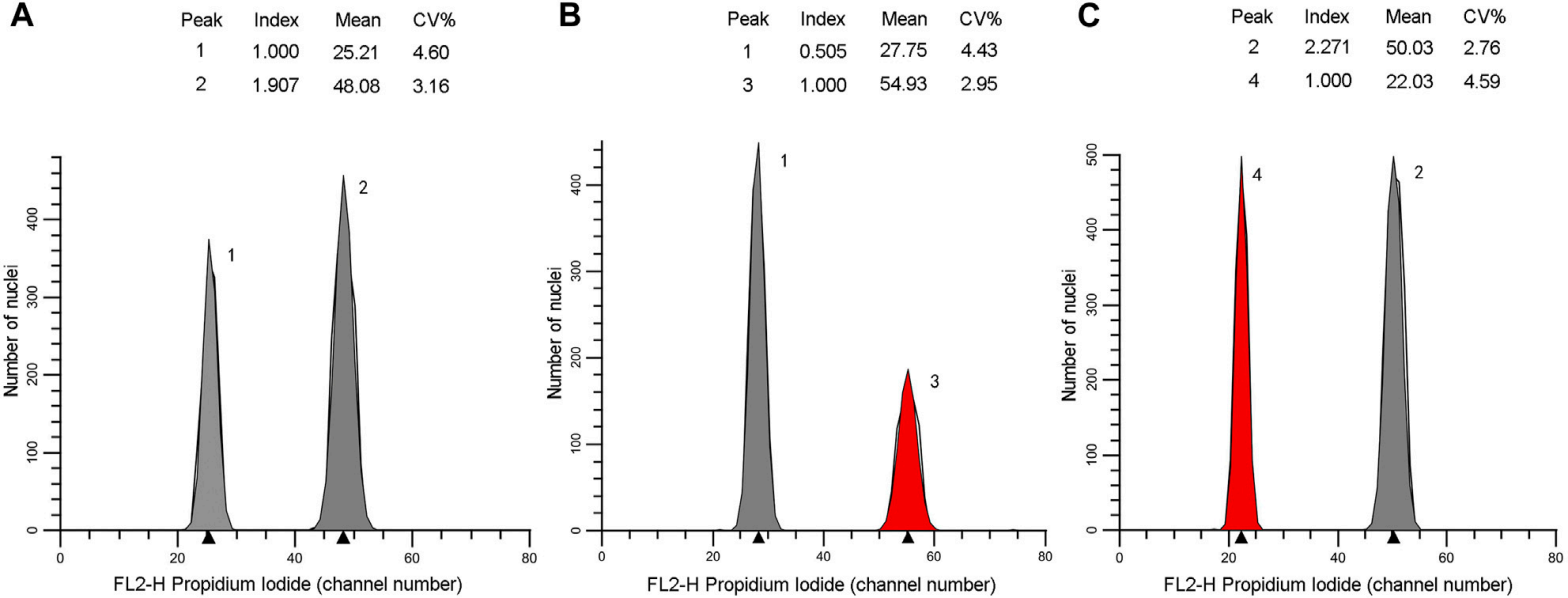
Flow cytometric histograms of the individuals N3-8 **(A,B)** and S1-2 **(A, C)**. *Xanthocyparis vietnamensis* (peak 1 and 2), standard I: *Pseudolarix amabilis* (peak 3), standard II: *Cupressus funebris* (peak 4).

The genome size estimates for the 16 individuals ranged 25.2–50.48 pg/2C. They could be distributed into two classes: Class 1 of lower values (25.42–26.50 pg/2C, mean = 26.08 pg/2C) corresponded to diploid with 2*n* = 2x = 22 for the individuals belonging to the northern populations, class 2 possessed higher values (46.59–50.48 pg/2C, mean = 48.02 pg/2C) corresponded to tetraploid with 2*n* = 4x = 44 for the individuals belonging to the southern populations (Table 2). Flow cytometric histograms obtained from analysis of two representative individuals, one each from the northern (N3-8) and southern (S1-2) populations, were shown in Figure 6.

## Discussion

### Genetic Diversity of *X. vietnamensis* in Southwestern China

Our population genetic survey of *X.vietnamensis* revealed that its populations from southwestern China exhibit lower diversity (mean value across all sampled populations: *H*_E_ = 0.45, *H*_O_ = 0.49) than the long-lived perennial (*H*_E_ = 0.68, *H*_O_ = 0.63), widespread (*H*_E_ = 0.62, *H*_O_ = 0.57), regional (*H*_E_ = 0.65, *H*_O_ = 0.65) and narrow range plant populations (*H*_E_ = 0.56, *H*_O_ = 0.52), but exhibit higher diversity than endemic plant populations (*H*_E_ = 0.42, *H*_O_ = 0.32) according to the mean values provided by Nybom (2004) based on microsatellite markers. Compared with other threatened species in Southern China and adjoining areas, the diversity of *X. vietnamensis* (*H*_E_ = 0.51, *H*_O_ = 0.45) was lower than that of *C. macrolepis* (*H*_E_ = 0.64, *H*_O_ = 0.71, 291 individuals) (Liao et al., 2015) and *F. hodginsii* (*H*_E_ = 0.64, *H*_O_ = 0.52, 427 individuals) (Yin et al., 2018), yet higher than that of *Glyptostrobus pensilis* (*H*_E_ = 0.27, *H*_O_ = 0.32, 343 individuals) (Wu et al., 2020), *Cathaya argyrophylla* (*H*_E_ = 0.41, *H*_O_ = 0.37, 49 individuals) (Wang et al., 2010) and *Abies ziyuanensis* (*H*_E_ = 0.44, *H*_O_ = 0.32, 139 individuals) (Tang et al., 2008). However, although the 33 individuals sampled across the six small populations in this study represent the majority of the known distribution of *X. vietnamensis* in southwestern China, limited sampling still may have led to an underestimation or inaccurate estimation for the genetic diversity of this species. Moreover, our loci number may be insufficient and the population indices in Table 4 should be treated with caution.

Some relict species endemic to South China were cold-adapted, for example, *C. argyrophylla* and *A. ziyuanensis* were restricted to a few isolated alpine habitats with cold microclimates. These species tend to have lower levels of genetic variation and are faced with a greater threat of losing their last refuge in South China in the face of global warming (Tang et al., 2008). In contrast, *X. vietnamensis* is a heliophile and subtropical or tropical plant (Averyanov et al., 2002), similar to *C. macrolepis* and *Pinus kwangtungensis* (Tian et al., 2008), there may be a wider range of potential habitats (including non-karst montane areas) in warm South China and adjacent regions in southeast Asia. But actually it is currently inhabiting isolated mountain tops, cliffs or slopes confining within karst areas and has a very small population size that might have been partly caused by historical deforestation (Zeng et al., 2007; Wang 2011; Jiang et al., 2014). At the same time, we detected a significant bottleneck event for *X. vietnamensis* populations in southwestern China, when assuming two different pre-bottleneck effective population size, suggesting that this species may have experienced a strong bottleneck event in the past. This provides reasonable explanation for the low-level genetic diversity of *X. vietnamensis*.

### Moderate Level of Population Differentiation and Significant Genetic Structure

Our population genetic survey revealed moderate genetic differentiation (F_ST_ = 0.125) among *X. vietnamensis* populations in southwestern China. This is similar to the mean values of anemochores (0.13) (Nybom, 2004), lower than those of *F.hodginsii* (0.157) (Yin et al., 2018), *C. macrolepis* (0.163) (Liao et al., 2015), *A. ziyuanensis* (0.209–0.250) (Tang et al., 2008), and *G. pensilis* (0.452, some populations are plantations) (Wu et al., 2020) in a similar geographic region. Varied topography of mountain regions in the tropic and adjacent areas tended to harbor Tertiary relict species and subdivide some species into isolated and divergent populations, which may have evolved independently during late Tertiary climate changes and Quaternary climate fluctuations (Hewitt, 2000; Tang et al., 2018). The karst landscape in Guangxi is a residual carbonate hill protruding from a surrounding corrosion plain (Gutiérrez and Gutiérrez, 2016). The hills are generally only hundreds of meters in height, and the nearby hill tops are generally hundreds of meters apart. Even so, the karst forest ecosystem here exhibits heterogeneous geomorphology and variegated vegetation (Su et al., 2017; Guo et al., 2018). The heterogeneous geomorphology may have contributed the genetic differentiation among populations of *X. vietnamensis*.

At the same time, we detected significant population genetic structure in *X. vietnamensis*, as both STRUCTURE and DAPC analyses clearly revealed a distinct division between the northern and southern population. In addition, a relatively strong barrier to gene flow between the northern and southern populations was identified by BARRIER analysis, and the estimated *N*_m_ from south to north or vice versa (Table 6) was limited, being consistent with the moderate genetic differentiation (Slarkin, 1985). This is supported by the fact that, there are non-karst areas between or among the karst landforms, which bear different ecological environments and interrupt the continuity of karst flora (Chen et al., 2012). For example, the Youjiang Basin, which mainly consists of broad non-karst terrains (Lu et al., 2006), is located at the midpoint between the northern and southern populations of *X. vietnamensis* (Figure 1). The Youjiang Basin and other non-karst areas between northern and southern population of *X. vietnamensis* might have acted as genetic barriers and resulted in isolated and fragmented populations, and restricted gene flow and migration between the northern and southern populations of *X. vietnamensis*.

Generally, bottleneck effects are found in populations with reduced population size due to natural catastrophes, habitat loss, alteration and fragmentation. As such, it is likely a historical climate event in combination with the fragmented habitat in this karst landscape may have led to the significant bottleneck detected in the *X. vietnamensis* populations. Meanwhile, in the context of demographic bottleneck, the isolation and fragmentation could have serious negative impact on the *X. vietnamensis* population due to random drift and inbreeding, and may have promoted genetic differentiation among populations (Nei et al., 1975; Sękiewicz et al., 2015).

### Variation in Genome Size and Asymmetric Gene Flow

Our genome size data showed that *X. vietnamensis* is mixed-ploidy and populations in Guangxi possess both diploid and tetraploid cytotypes. The average tetraploid genome size of 48.02 pg/2C was similar to but slightly larger than previous study (44.60 pg/2C) using another internal standard and nuclei isolation buffers (Farhat et al., 2021); the average diploid genome size of 26.08 pg/2C is similar to that of diploid *Juniperus* species (Farhat et al., 2019). Although only four representative individuals per population were sampled from two populations each from northern and southern population groups, we found that all eight individuals from northern populations are diploids and these from southern populations are tetraploids.

While there was significant difference in genome size between the northern (diploid) and southern (tetraploid) populations, microsatellite diversity varied little between them. These differences may reflect the recent origin of tetraploid from the diploid (Lumaret and Barrientos, 1990; Ickert-Bond et al., 2020). However, these two cytotypes might have distinctly different genetic structure (Figures 3, 4), which was probably due to habitat differences and spatial isolation (Laport et al., 2016). Tetraploid populations appear to have greater negative inbreeding coefficients (*F*_IS_) (Table 4). We tested the impact of small population size on *F*_IS_ (Supplementary Table S2), and found a trend of negative *F*_IS_ with greater absolute values when sample size is small, but smaller size did not cause a shift of *F*_IS_ from positive value to negative value or the other way round. These results suggest seedling production by selfing in tetraploid populations of *X. vietnamensis*. As reported in previous studies, chromosome doubling can lead to a breakdown of self-incompatibility (Soltis and Soltis, 2000; Sutherland et al., 2018). Thus, tetraploid populations may be more likely to establish after expansion or dispersal than diploid populations. As far as we know, many more tetraploid individuals and populations were found in the neighboring areas——Vietnam (Averyanov et al., 2013; Farhat et al., 2021). Our data revealed historical gene flow from north (diploid population) to south (tetraploid population) was higher than the reverse (Table 6). It is likely that tetraploid *X.vietnamensis* in southern population may be of autopolyploid origin and diploids in the northern populations may be the source populations, yet these hypotheses need to be further tested.

### Conservation Implications

There is clear genetic and ploidy level distinction between the north and south populations in southwestern China according to the STRUCTURE analysis and genome size analysis. The disjunct geography and topography of the karst mountains and over 300 km of geographical distance between the northern and southern populations may have acted as barriers to gene flow. Hence the northern and southern populations should be treated as two different management units. Populations of *X. vietnamensis* are isolated as their habitat is confined to the mountain peaks. To reduce inbreeding and minimize bottleneck effect, artificial pollination or transplanting among fragmented populations in each unit should be implemented. Considering the rarity of wild individuals at present, we suggest *in situ* artificial regeneration programmes immediately for the populations, and urgent trials of *ex situ* plantations in Guangxi, China, through the collection, germination, cultivation of seeds and twig cuttage, similar to the strategy that has proven successful in Vietnam for this species.

In our study, sampled populations were restricted to provincial or national borders, remote from the human residential belt. The regions between the northern and southern populations are substantially impacted by humans (Zeng et al., 2007; Wang, 2011; Jiang et al., 2014). These imply that human activities led to the loss of habitats and the extremely small population size of *X. vietnamensis*. The northern populations are mostly located in the Guangxi Mulun National Nature Reserve, and the southern populations are located in the Guangxi Laohutiao Provincial Nature Reserve. In general, management effectiveness of nature reserves in China are relatively good, especially those with higher management level; but there are still some problems of nature reserves in China, such as the lacking information of boundaries and tenures, the poorly organized institution, the lacking of staff, fund and so on (Wang and Li, 2021). The Laohutiao Provincial Nature Reserve stretches 69 km along the border of China and Vietnam. Generally, it is not easy to effectively protect the ecology and biodiversity in the national borders (Wang and Li, 2021). Unfortunately, the distribution area of the southern populations of *X. vietnamensis* was accessible for agricultural exploitation, as we saw during the field survey. We recommend more attention and tighter management for the conservation of forest trees in this reserve.

## Data Availability

The datasets presented in this study can be found in online repositories. The names of the repository/repositories and accession number(s) can be found below: GenBank, MZ514645-MZ514664; Dryad doi:10.5061/dryad.qnk98sfh7.
