# Supplementary material for: Distinct Genetic Structure Reflects Ploidy Level Differentiation in Newly Discovered, Extremely Small Populations of Xanthocyparis vietnamensis from Southwestern China
Source: Front Genet. 2021 Nov 1;12:733576. doi: 10.3389/fgene.2021.733576 (PMC8591046; doi:10.3389/fgene.2021.733576)
Supplement: Supplementary file 1 [file Table1.docx]

Journal: Frontiers in Genetics

Title: Distinct Genetic Structure Reflects Ploidy Level Differentiation in Newly Discovered, Extremely Small Populations of *Xanthocyparis vietnamensis* from Southwestern China

Authors: Yuliang Jiang, Tsam Ju, Linda E. Neaves, Jialiang Li, Weining Tan, Yusong Huang, Yan Liu and Kangshan Mao

**Table S1** Characteristics of the 20 microsatellite markers developed for *Xanthocyparis vietnamensis.*

| Locus | Primer sequences (5'-3') | Repeat motif | Product size (bp) | Tm(℃) | Fluorescent dye | GenBank accession no. | Protein |
| --- | --- | --- | --- | --- | --- | --- | --- |
| seq89 | F: ACCCACTTTCTCGTGCTTGT  R: GCCATGGAGTTCGAAACTGT | (AG)7 | 324 | 60 | FAM | MZ514645 | K4CJJ1 (NCED2_SOLLC), 9-cis-epoxycarotenoid dioxygenase NCED2 |
| seq2225 | F: GACTCCCATCAAATTCTTCAAA  R:GACTGCTGCAAAGACTCGTG | (AC)7 | 257 | 58 | HEX | MZ514646 | Unknown |
| seq24651 | F: ATTCCCCATTCAAAGTGCAG  R:GGGTATTCCCTCTCAAAGGC | (TG)10 | 157 | 60 | TAMRA | MZ514657 | Unknown |
| seq5172 | F: GGCTTATTTTCCCAAGAGGG  R:TCCTTTGCATATCAAAAACCAA | (TG)10 | 273 | 60 | HEX | MZ514647 | Unknown |
| seq11372 | F: CAGGGACGAGCACAAGTACA  R:TTGCATTAAGCCACCTTCAA | (GT)7 | 214 | 60 | HEX | MZ514648 | Unknown |
| seq12084 | F: GCCTGACTGTGAAAAGCTCC  R:GGTTGCACGAAACGAAACTT | (TA)7 | 380 | 60 | FAM | MZ514649 | Unknown |
| seq13330 | F: GGGAGTACATCATCCGCTGT  R:GGGATAATGTCTAGGCCAACC | (AT)10 | 320 | 60 | FAM | MZ514650 | Unknown |
| seq17433 | F: TCACAAAGAAATGGCGAGTG  R:GTGCATGCATACGTAGGTGG | (AC)11 | 371 | 60 | HEX | MZ514651 | Unknown |
| seq19571 | F: AGATTTGGCTGGGCTCCTAT  R:TCACTGACAATCTTGACGGC | (CGC)5 | 276 | 60 | HEX | MZ514652 | Q84QC2 (ERF17_ARATH), Ethylene-responsive transcription factor ERF017GO:0000976 |
| seq20427 | F: AGCCTTGCAGTTTTCCTCAA  R:AGGGTCGACAGAGCTTGAAA | (AT)6 | 330 | 60 | FAM | MZ514653 | Q9CAG5 (PUB7_ARATH), U-box domain-containing protein 7GO:0004842 |
| seq22472 | F: ACAGGAAGCCAAAGGAAGGT  R:TTAAGCCCAATGGTGAAAGG | (AAT)5 | 258 | 60 | HEX | MZ514654 | Q9SIT7 (PP151_ARATH),Pentatricopeptide repeat-containing protein At2g13600GO:0005739 |
| seq22970 | F: GGTTATGACAAGGGGGTGG  R:CCTTTTCCTCTCCCCCTCTA | (AG)6 | 323 | 60 | FAM | MZ514655 | Unknown |
| seq24226 | F: CCCAACCCTCTAATTCGTGA  R:AATTGTGAAAGCCAAATGGG | (AG)6 | 352 | 60 | FAM | MZ514656 | A2YQ56 (LONM_ORYSI), Lon protease homolog |
| seq25166 | F: TTTCATGTGCTGTTCCTTAGACA  R:TGAGTCTTGGGGATAGGGTG | (GT)16 | 135 | 60 | TAMRA | MZ514658 | Unknown |
| seq25799 | F: CATGCTCGTAAGACTGCATGA  R:TGGATTGGCTTGATGACTGA | (ATA)8 | 149 | 60 | TAMRA | MZ514659 | Unknown |
| seq27386 | F: TTATACGCCTGCATGCAAAA  R:TTCCCTCTTCCTCAATCCCT | (AC)6 | 315 | 60 | TAMRA | MZ514660 | Unknown |
| seq30772 | F: GGCAGGAGTCAGGTCAAGTC  R:GCTTGTGCATTTGTGCAGTT | (CA)10 | 133 | 60 | TAMRA | MZ514661 | Unknown |
| seq31782 | F: TCTGCACTGACGCCTAACAC  R:ACTCAGCTGCTGCCACTCTT | (GA)8 | 358 | 60 | FAM | MZ514662 | Unknown |
| seq33197 | F: TCCTTCTCTTCTCCCCCTTC  R:TTGTAACCCTAGGATCCCCC | (TC)6 | 135 | 60 | TAMRA | MZ514663 | Unknown |
| seq34736 | F: AGCTGTCAGGCCAGTTTGTT  R:ACTCATGAGGAGGATGTGGC | (TTC)13 | 213 | 60 | HEX | MZ514664 | Unknown |
