# Supplementary material for: Distinct Genetic Structure Reflects Ploidy Level Differentiation in Newly Discovered, Extremely Small Populations of Xanthocyparis vietnamensis from Southwestern China
Source: Front Genet. 2021 Nov 1;12:733576. doi: 10.3389/fgene.2021.733576 (PMC8591046; doi:10.3389/fgene.2021.733576)
Supplement: Supplementary file 2 [file Table2.docx]

Journal: Frontiers in Genetics

Title: Distinct Genetic Structure Reflects Ploidy Level Differentiation in Newly Discovered, Extremely Small Populations of *Xanthocyparis vietnamensis* from Southwestern China

Authors: Yuliang Jiang, Tsam Ju, Linda E. Neaves, Jialiang Li, Weining Tan, Yusong Huang, Yan Liu and Kangshan Mao

**Table S2** Population genetic indices of four and five individuals from subpopulation N3, N4, S1 and S2.

|  | *n* | *A* | *A*_E_ | *I* | *H*_O_ | *H*_E_ | *F*_IS_ |
| --- | --- | --- | --- | --- | --- | --- | --- |
| N3 | 4 | 2.2 | 1.8 | 0.585 | 0.350 | 0.361 | 0.051 |
|  | 5 | 2.9 | 2.1 | 0.740 | 0.335 | 0.413 | 0.136 |
| N4 | 4 | 2.4 | 2.0 | 0.616 | 0.425 | 0.367 | -0.166 |
|  | 5 | 2.6 | 2.0 | 0.691 | 0.433 | 0.404 | -0.055 |
| S1 | 4 | 2.7 | 2.1 | 0.732 | 0.563 | 0.427 | -0.324 |
|  | - | - | - | - | - | - | - |
| S2 | 4 | 2.0 | 1.8 | 0.556 | 0.613 | 0.370 | -0.677 |
|  | 5 | 2.5 | 2.0 | 0.684 | 0.610 | 0.410 | -0.452 |
