# Supplementary material for: Distinct Genetic Structure Reflects Ploidy Level Differentiation in Newly Discovered, Extremely Small Populations of Xanthocyparis vietnamensis from Southwestern China
Source: Front Genet. 2021 Nov 1;12:733576. doi: 10.3389/fgene.2021.733576 (PMC8591046; doi:10.3389/fgene.2021.733576)
Supplement: Supplementary file 3 [file Image1.pdf]

Journal: Frontiers in Genetics

Title: Distinct Genetic Structure Reflects Ploidy Level Differentiation in Newly Discovered, Extremely Small Populations of *Xanthocyparisvietnamensis* from Southwestern China

Authors: Yuliang Jiang, Tsam Ju, Linda E. Neaves, Jialiang Li, Weining Tan, Yusong Huang, Yan Liu and Kangshan Mao

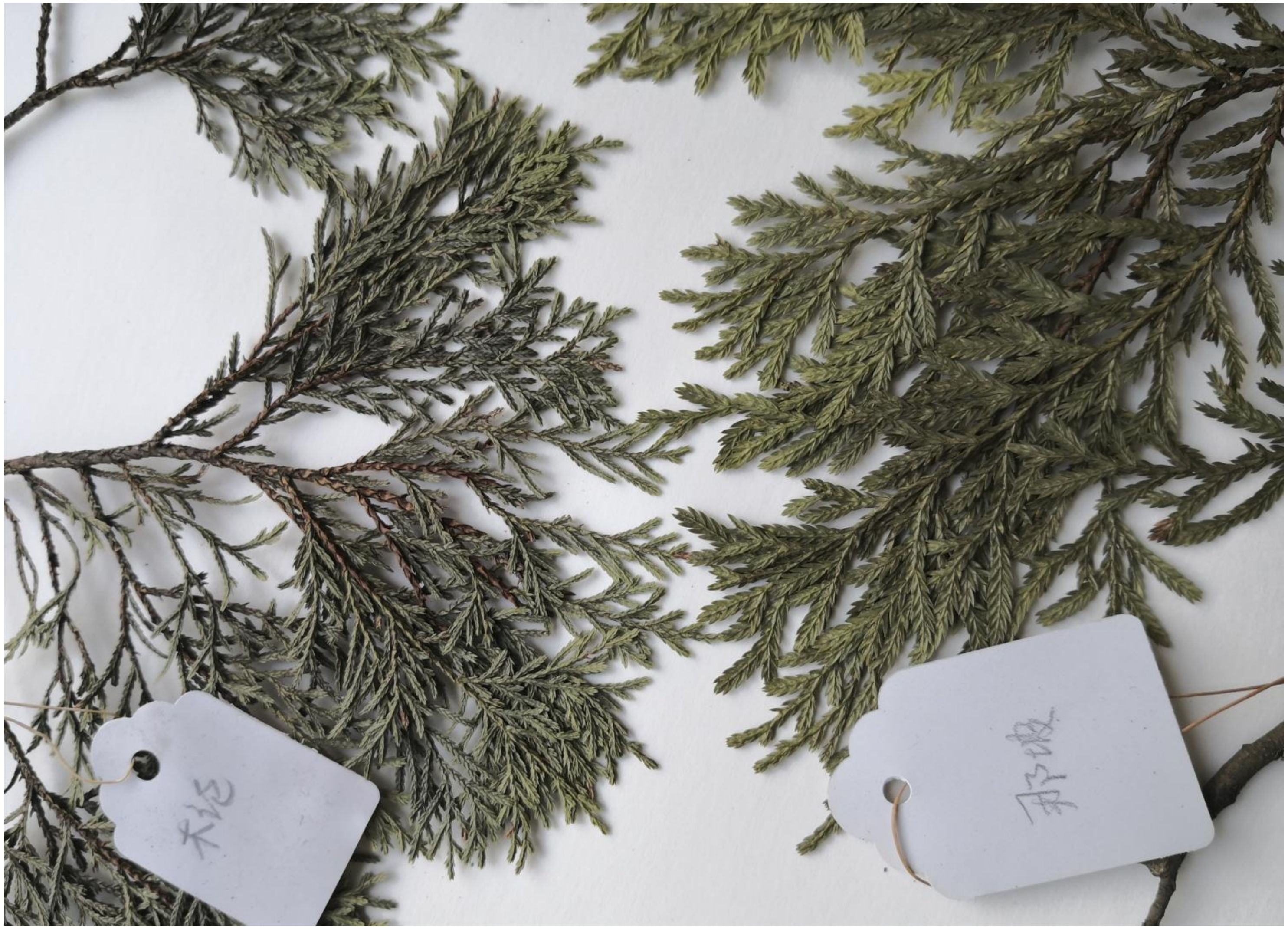

Fig. S1 Specimens of *Xanthocyparisvietnamensis*from northern population (left, signing "木论") and southern population (right, signing "那坡") differ in the size and shape of branchlets with scale-like leaves.
